# Silver nanowire-based transparent, flexible, and conductive thin film

**DOI:** 10.1186/1556-276X-6-75

**Published:** 2011-01-12

**Authors:** Cai-Hong Liu, Xun Yu

**Affiliations:** 1 Department of Mechanical and Industrial Engineering, University of Minnesota Duluth, Duluth, MN 55812, USA

## Abstract

The fabrication of transparent, conductive, and uniform silver nanowire films using the scalable rod-coating technique is described in this study. Properties of the transparent conductive thin films are investigated, as well as the approaches to improve the performance of transparent silver nanowire electrodes. It is found that silver nanowires are oxidized during the coating process. Incubation in hydrogen chloride (HCl) vapor can eliminate oxidized surface, and consequently, reduce largely the resistivity of silver nanowire thin films. After HCl treatment, 175 Ω/sq and approximately 75% transmittance are achieved. The sheet resistivity drops remarkably with the rise of the film thickness or with the decrease of transparency. The thin film electrodes also demonstrated excellent flexible stability, showing < 2% resistance change after over 100 bending cycles.

## Introduction

Transparent conductive thin film electrodes are widely used for liquid crystal displays (LCDs), touch screens, solar cells, and flexible displays [1,2]. Among these applications, the most commonly used materials are doped metallic oxides, mainly indium tin oxide (ITO) because of their high electrical conductivity and high optical transparency. However, there are many drawbacks in the case of ITO transparent electrodes. They are prone to cracking on flexible substrates. In addition, the ITOs are costly and require high temperature during thin film fabrication processes [3]. However, the future display and other optic-electronic devices will require suitable methods for flexible transparent electrodes to be produced at low cost and in a large scale, such as roll-to-roll coating or ink-jet printing method. The substitutes for ITO are required for the developing electronic industry, and the search for such materials is mainly focused on conductive polymers and conductive nano-structures with high aspect ratio, whereas the low conductivity of polymer transparent electrode (approximately 1 S/cm) [4] restricts their applications. In recent years, 1D nano-structures have been actively researched as candidates for future transparent electrode materials, including nanowires, nanotubes, and nanorods [2,5-8]. Among these, carbon nanotubes are extensively explored in the past few years because of their theoretically ballistic conductivity, good electromechanical properties, and chemical inertness [8,9]. The ID nature of these nanostructures leads to increased optical transparency compared to a continuous, 3D material. However, nanotube films have yet to match the properties of ITO continuous films (i.e., transmittance = 90%, and sheet resistance < 100 Ω/sq), because nanotube film performances are hampered by the inevitable defects on the tubes, bundling between tubes, and the mixture of metallic and semiconducting carbon nanotubes [7]. On the other hand, silver (Ag) nanowire (NW) is another promising alternative, and it has been reported to have the potential to surpass and replace ITO [10,11].

Silver nanowires have been attracting more and more attention because of their intriguing electrical, thermal, and optical properties [12]. Silver has the highest electrical conductivity (6.3 × 10^7 ^S/m) among all the metals, by virtue of which Ag NWs are considered as very promising candidates in flexible electronics. Lee et al. [11] have pioneered this material and shown that the cast Ag NW thin film used as transparent electrode showed equal merit or better than that as compared with sputter-coated ITO in solar cells. The gold/silver alloy NWs were also synthesized and studied as transparent electrode material by Azulai et al. [6]. Since then, Ag NW films have been fabricated using techniques, such as vacuum filtration, transfer printing onto poly(ethylene terephthalate) (PET) substrates [13], drop casting [11,14], and air-spraying [15] from NW suspension. The vacuum filtration, the so-called transfer method, produces highly transparent films with excellent conductivity [13], but the films possess irregular morphologies and significant roughness [16]. Moreover, the process is not scalable. Using drop casting method always shows coffee rings and discontinuous film on the substrates. The film obtained from air-spraying coating is much better, but still forms sparse and non-uniform networks. In brief, most of the processes proposed so far cannot be ported easily to large scale production. Moreover, the researches on the film properties and effect factors have been very limited. In this article, we demonstrate uniform and transparent Ag NW film on plastic substrate via a scalable, simple, and low-cost process (i.e., rod coating), and means to improve the performance of flexible electrode, i.e., HCl treatment and protection coating.

## Materials and experimental method

### Materials and film fabrication

**Figure 1:**
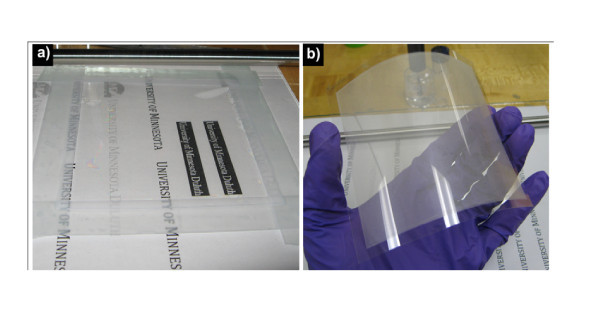
**Pictures of the coating apparatus and fabricated Ag NW thin film**. **(a) **Wire-wound rod (Meyer rod)-coating apparatus for Ag NW thin film. A piece of PET film was stuck on glass board with scotch tape before roll coating. **(b) **A large, uniform, and flexed Ag NW film on PET substrate with dimension of about 80 mm × 100 mm.

**Figure 2:**
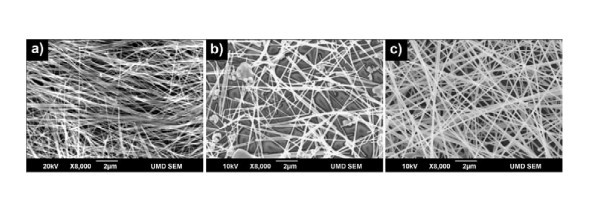
**SEM images of as-purchased Ag NWs with different sizes**. **(a) **1#: *d_t _*= 66 nm, *L *= 7.4 μm; **(b) **2#: *d_t _*= 102 nm, *L *= 15 μm; **(c) **3#: *d_t _*= 122 nm, *L *= 34 μm.

Silver nanowires were purchased from Seashell Technologies (La Jolla, CA, USA) as suspension in isopropyl alcohol with concentrations of 25 mg/ml. A small volume of dispersion was diluted down to approximately 1 mg/ml with isopropyl alcohol. This was subjected to half-an-hour sonication in a sonic bath. Then, this suspension was applied to a 50 mm × 100 mm poly(ethylene terephthalate) (PET) substrates by a manually controlled wire-wound rod (Meyer rod 10# and 20#), i.e., pushing the suspension on top of the substrate with a Meyer rod. Figure 1a shows the roll-coating set-up. After drying in air at room temperature, additional Ag NW layers could be coated above the initial Ag NW layer if needed. Examples of the transparent films are shown in Figure 1b. The Ag NW film looks very uniform over the entire substrate. In this study, three Ag NW samples with different sizes were used (SEM images shown in Figure 2), marked as 1# (*d_t _*= 66 nm, *L *= 7.4 μm), 2# (*d_t _*= 102 nm, *L *= 15 μm), and 3# (*d_t _*= 122 nm, *L *= 34 μm). From sample 1# to 3#, both the diameter and length of nanowires are in the ascending order of increase.

To improve the conductivity of Ag NW film, concentrated HCl was employed to destroy the oxidation layer on the NW surface. Ag NW thin films on PET substrate was incubated in the HCl vapor (20 to 60°C) volatilized from concentrated HCl for 5-10 min. The prepared NW films are easy to scratch and damage due to the weak adhesion to PET substrate. For protecting the prepared transparent electrodes and prolonging their lifetime, a protective layer is required. Several drops of colorless nail polish liquid (commercial products) were cast on the film surface. Then, a slim glass vial or Meyer rod was rolled to the other end of the electrode to form a uniform nail polish layer. After being placed in the air for 1 min at room temperature, the nail polish formed a solid and thin protective layer on the Ag NW film. No observable scratch or change on Ag NW layer was found during the coating of nail polish layer.

### Optical transmittance measurement and SEM

All the SEM images were taken using a JEOL JSM-6490LV SEM. The Energy Dispersive Spectrometer (EDS) was utilized to examine the approximated chemical compounds for the Ag NWs before and after the HCl acid vapor treatment/incubation. Optical transmission spectra of the Ag NW films were recorded using a UV-Vis-NIR spectrometer (Jasco V-670) without using integrating sphere, with a sheet of PET being used as the reference. Sheet resistance measurements were made using the four-probe technique with a Keithley 2701 source meter. Bending experiments were performed by the two-point bending test in which a piece of Ag NW film on PET was bent into nearly a circle which was constrained manually. Then, the sheet resistance was measured using a digital multimeter.

## Results and discussion

Figure 1b shows a Ag NW film on a PET substrate fabricated via wire-wound rod coating. Draw-down rod coating is a well-known coating technique widely used by industrial laboratories for making thin films in a continuous and controlled manner. The liquid that can be coated effectively by the Meyer rod method can then be readily adapted to more controllable, higher throughput methods, such as slot, slide, and roll-to-roll coating [17]. Different from the carbon nanotubes film previously obtained from rod-coating technique [18,19], Ag NW suspension can be directly and uniformly coated onto PET at room temperature without any hydrophilic or hydrophobic pre-treatment of PET or coating materials, and no surfactants are required. However, the solvents are found to be very important to the uniformity of films. We have dispersed Ag NW in distilled water, surfactants aqueous, ethylene glycol (EG), methanol, and isopropanol. After 1-h sonication, all of these suspensions agglomerated in 5 h. On the other hand, Ag NWs show much better dispersibility, and are easier to re-dispersion in methanol and isopropyl alcohol than in aqueous solution. It is found that isopropyl alcohol leads to the most uniform coatings. The samples illustrated in this article were all from Ag NW isopropyl alcohol suspensions. Dan et al. [17] have illustrated that the uniformity of the films obtained from rod coating were determined by the surface tension and viscosity of liquid, which means that the groove size in wire-wound rod, solvents evaporation, concentration, and dispersibility of suspension, interactions between nanomaterial and substrates all impact the film homogeneity. In this case, because of the good evaporation of isopropanol, the films show line recession when the concentration of Ag NWs reaches above 5 mg/ml. For the same NW suspension, 10# rod is fabricated with better coating than 20# rod.

**Figure 3:**
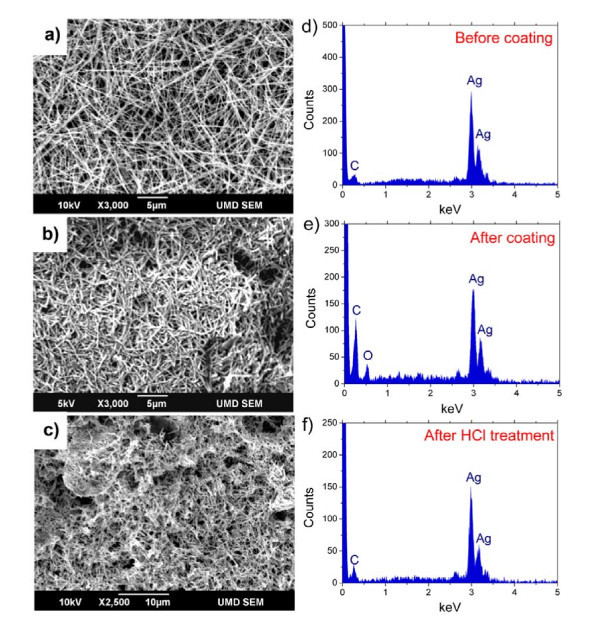
**SEM images and EDS spectra for Ag NW, respectively, before coating (a) (d), after coating (b) (e), and after intensive HCl treatment (c) (f)**.

The original Ag NW thin films fabricated are non-conductive (*R *> 20 MΩ) even when the transmittance of the film is less than 30%. This is because of the oxidation of the NW surface when most of the films were exposed in the air during the coating and drying process. In this study, concentrated HCl is employed to dwindle the oxidation on the Ag NW surface. A piece of Ag NW thin film on PET substrate was incubated in the HCl vapor (20 to 60°C) for approximately 10 min. The resistance dramatically reduced to below 500 Ω. It can be seen from SEM images that the nanowires become thinner and shorter after intensive HCl treatment, whereas there is no shortening effect with a milder reaction (Figure 3a, b, c).

The HCl treatment destroys the oxidation layer on the nanowire surfaces and improves the contacts between NWs, thus increasing the film conductivity and transparency. To detect the element distribution variation during these processes, the EDS with a large spot size (approximately 1 μm) was performed (Figure 3d, e, f). In these EDS spectra, we can see a clearly intensity change of oxygen element peak (at around 0.5 keV). In Figure 3d, there is no appearance of "O" peak in the EDS spectra of the drop-cast sample with very good conductivity, from a very concentrated Ag NW suspension. However, after the rod-coating procedure, the content of oxygen distinctively increased (Figure 3e). Then, the oxidation layer was effectively removed by the HCl treatment, as shown in Figure 3f. During the process, the silver oxide (e.g., AgO, Ag_2_O, Ag_3_O_2_, or Ag_4_O_3_) reacted with HCl to form AgCl, and then the AgCl was partially photodecomposited under light illumination. However, Cl species is one of the main impurity during synthetic process [12], and nearly all the samples show very small amount of chlorine (Cl_K _at approximately 2.65 keV). There is no obvious increase on the amount of Cl in sample after HCl treatment, implying an efficient photodecomposition.

Cui et al. [10] reported that the junction resistance between the Ag NWs is larger than 1 GΩ. If the surfaces of Ag NWs are oxidized, then both the single NW and the NW-NW junction resistances would increase because of the semiconducting property of silver oxide, especially the junction resistance. From the EDS spectra and film resistance test, the HCl treatment effectively reduces the "O" abundance in the film and the contact resistance between Ag NWs. Tests show that there is nearly no change in the resistance of HCl-treated thin film staying in air. This is most likely because the inner NW layer and NW contact points are not re-oxidized. Further optimization on HCl process will allow for better performance as a result of transparent, flexible electrode. The HCl treatment is an easier approach for improving the thin film conductivity than the reported method in the recent literature which employed Ag-Au core-shell NWs to replace Ag NWs [10]. Other simple procedures also may help to improve the conductivity of film fabricated by rod-coating methods, such as H-plasma treatment and incubating/annealing in reducing atmosphere, etc.

**Figure 4:**
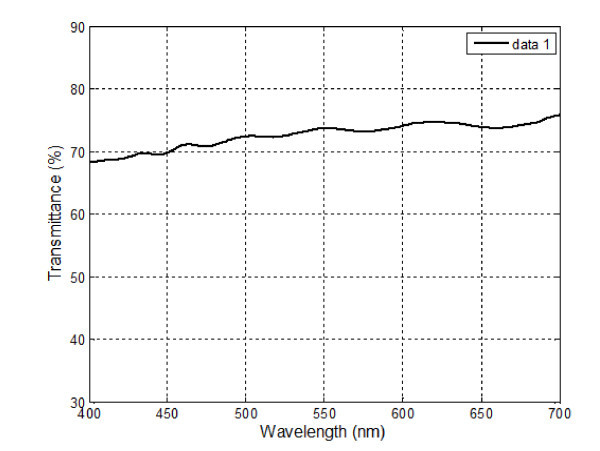
**Light transmittance spectrum of the Ag NW thin film in the visible light region**.

A typical optical transmission spectrum in visible light region is shown in Figure 4 for a Ag NW thin film with surface resistivity of 170 Ω/sq, which indicated an average transmittance of approximately 74%. From transmission spectra, it can be seen that Ag NWs do not have any characteristic absorption peaks except of periodic intensity oscillation which is a feature for the light reflection of uniform thin film [20]. Because the diameters of Ag NWs or NW bundles are large (approximately 100 nm), the reflection and scattering of light on NW film increase, and hence, decrease the film transmittance. However, reflection and scattering benefit the application of Ag NW films in photovoltaic devices [10,14], in which the Ag NW electrode-contained solar cells show larger photocurrent than the one using ITO film as electrode. As in other electronic devices, film with small diameter NW will show better transparency at same conductivity. Coating polymeric anti-reflective material over the Ag NW networks also could largely lower the light reflection.

**Figure 5:**
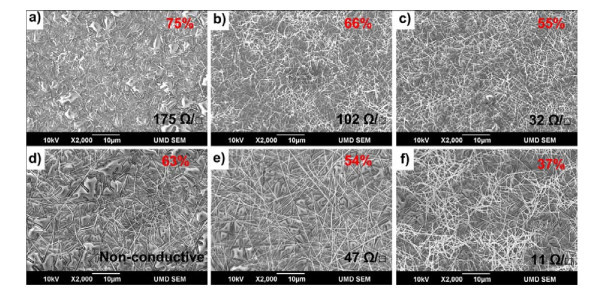
**SEM images of Ag NW films with different thickness**. **(a-c) **SEM images of the surface of films with increasing thickness from NW sample 1# and **(d-f) **films made from NW sample 3#. The corresponding optical transmittances at 550 nm and sheet resistivity of thin films are indicated on the images: (a) 175 Ω/sq, *T *= 75%; (b) 102 Ω/sq, *T *= 66%; (c) 32 Ω/sq, *T *= 55%; (d) non-conductive, *T *= 63%; (e) 47 Ω/sq, *T *= 54%; (f) 11 Ω/sq, *T *= 37%.

Ag NW films with varied thicknesses from different samples are illustrated in Figure 5. For each of these samples, an array of films with different thicknesses by repeating the coatings and varying the NW concentrations in suspension has been prepared. Figure 5a, b, c and 5d, e, f shows successive SEM images with increasing film thickness (decreasing transmittance) for Ag NW 1# and 3# (see "Materials and experimental method" section), respectively. It can be seen from these SEM images that the one with relatively high transmittance has sparse NW networks. The bare and insulated PET substrates were charged and showed bright convex morphology. As the thickness was increased, the networks become dense, and the substrates appear less frequently. Drawing a side-by-side comparison between samples 1# and 3#, it can be found that the networks formed by the smaller NWs show 10-20% higher transmittance than the one composed of larger NWs even though the latter have similar network density. For Ag NW 3#, the NWs have much larger diameters with a range of 100-200 nm, and hence have much larger diffusion, scattering, and reflection which lead to lower transmittance. The film shown in Figure 5d possesses a low transmittance of approximately 63% even though there are large holes between NWs which subsequently make the film non-conductive. The shorter NWs have better transparency, while the longer one could lessen the required contact times and thus reduce the contact resistance which has been confirmed to be the main part of the sheet resistance. Hence, to improve the performance of Ag NW conductive films, NWs with small diameter and large aspect ratio are preferred. In addition, with the thickness or network density increasing, the uniformity of network appears to be improving.

It is known that the vacuum filtration method always produces transparent films with outstanding conductivity. It should be noted that the reported Ag NW transparent conductors prepared by vacuum filtration show better transparency and conductivity than the samples used in this study, which give a *Rs *= 13 Ω/sq for *T *= 85% as the best result in relation to the similar Ag NWs. The main reasons for the better conductivity are probably the better-dispersed Ag NWs as individual wires in the solid film and no oxidation in the contact area between the junction and the inner layer of NWs. However, vacuum-filtrated Ag NWs have weak adhesion to most substrates (e.g., PET, glass, Si, etc.) [13], making the transfer process much more challenging. Moreover, the vacuum filtration approach cannot meet the requirement of the scalable production. The excellent result from thin film produced via vacuum filtration method also implies that there is plenty of room for improving the performance of the transparent conductors fabricated by the scalable rod-coating.

**Figure 6:**
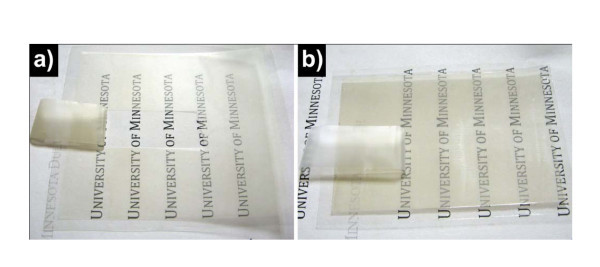
**Photograph of adhesion test with scotch tape on Ag NW film on PET substrate before (a) and after (b) coating with colorless nail polish as protective layer**.

To be applied in mass production, the transparent electrode must reach certain stability during the device fabrication and usage. However, one problem with the Ag NW transparent electrode is that the Ag NW thin film on PET plastic substrate is easy to peel off and be scratched. Therefore, it is necessary to protect the Ag NW electrode with a protective layer. Colorless nail polish was chosen in this study as the material of the protection coating, because it can dry in 1 min and is scratch resistant. Figure 6 shows respective photographs for adhesion test using 3 M scotch tape for the pristine-coated and nail polish-coated Ag NW films on the PET substrate. With pristine-coated Ag NW thin film, the Ag NW layer is easily detached from the surface of PET by the scotch tape as shown in Figure 6a. This suggests that the Ag NWs have poor mechanical adhesion to the plastic substrate. Whereas, the scotch tape did not peel off any Ag NWs on the nail polish coated Ag NW electrode (Figure 6b). Also no film cracking was observed after intensive bending. In addition, as shown in Table 1, the film shows nearly no increased resistance after being protected by nail polish, i.e., the protection of Ag NW film did not introduce additional resistance to the electrode. A small decline of the film resistance in the A and B films (shown in Table 1) can be attributed to the more compact Ag NW layer and better contact between Ag NWs after top coating. Although the little increase in film resistance of film C might be caused by the incautious shift between NWs during rolling down of the protective layer, there was no change in the NW layer under naked-eye observation.

**Table 1:** Resistance of Ag NW film before and after protective coating

Sample	*R *(Ω)	*R*_after protective coating _(Ω)
A	51.6	49.9
B	26.3	25.5
C	13.5	17.6

**Figure 7:**
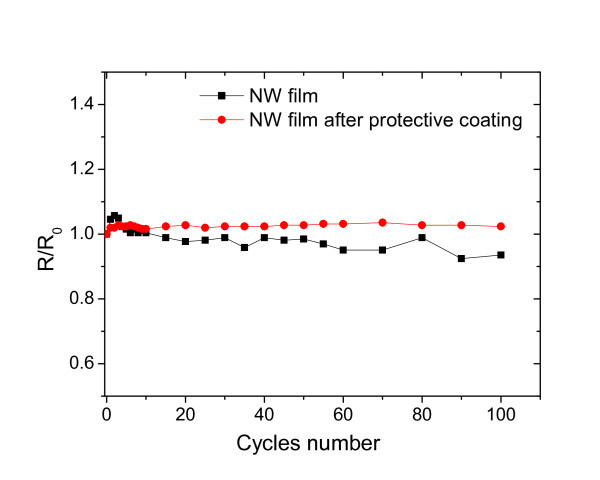
**Variations in resistance of Ag NW film as a function of the number of cycles after repeated tensile bendings to form nearly a circle**.

As mentioned earlier, poor flexibility of ITO film is one of its many drawbacks. The development of a high-performance transparent conductor that is flexible would remove a significant barrier to the development of low-cost flexible electronics. To test the flexibility of the Ag NW film conductor, bending tests were performed, and the representative result is illustrated in Figure 7. It is notable that the Ag NW film shows less than 2% deviation around the mean resistance during 100 cycles of bending. After 50 cycles, the sheet resistance exhibits even better stability, revealing a very high tolerance to bending.

## Conclusions

This research demonstrated Ag NW-based transparent and conductive thin films with excellent mechanical flexibility. The uniform and transparent thin films were fabricated via scalable, low-cost rod coating. With respect to the film uniformity, Meyer rod size, solvents, and concentration of NW suspension are the important factors. It was found that the simple HCl treatment can prominently decrease the thin film sheet resistance. SEM images and EDS spectra confirmed that the HCl treatment effectively eliminated the oxidation of nanowires and slight thinning of the wires, both of which could benefit the performance of NW film electrodes. The film transmittance and the sheet resistance as a function of film thickness were also studied. Electromechanical testing demonstrated that the film is extremely stable under bending with variation of sheet resistance being less than 2% over 100 bending cycles. Further treatment of thin film electrodes involved a performance-enhanced coating, i.e., a protective layer in this research. Using nail polish, NW films were successfully protected, and showed much better stability under both scratching and bending. Although the electronic and optical properties of the Ag NW thin film have not caught up with ITO yet, this proposed fabrication method is simple and easy to be scaled up for large films, which is one of the key issues for applying this technology to commercial applications. Moreover, this fabrication process also has the advantages of being low cost and with no need for expensive equipment. Therefore, it is believed that Ag NW will be a very promising candidate for transparent, flexible electrodes, especially in organic electronic devices and solar cells.

## Abbreviations

EDS: energy dispersive spectrometer; EG: ethylene glycol; HCl: hydrogen chloride; ITO:indium tin oxide; LCDs: liquid crystal displays; NW: nanowire; PET: poly(ethylene terephthalate).

## Competing interests

The authors declare that they have no competing interests.

## Authors' contributions

CHL participated in the experiment design, carried out the experiments, tested the thin films, and wrote the manuscript. XY designed the experiments and testing methods, and helped to draft the manuscript. All authors read and approved the final manuscript.
